# Resignation Syndrome: Catatonia? Culture-Bound?

**DOI:** 10.3389/fnbeh.2016.00007

**Published:** 2016-01-29

**Authors:** Karl Sallin, Hugo Lagercrantz, Kathinka Evers, Ingemar Engström, Anders Hjern, Predrag Petrovic

**Affiliations:** ^1^Centre for Research Ethics and Bioethics (CRB), Uppsala UniversityUppsala, Sweden; ^2^Department of Women’s and Children’s Health, Division of Neonatology, Karolinska InstituteSolna, Sweden; ^3^School of Health and Medical Sciences, Örebro UniversityÖrebro, Sweden; ^4^Centre for Health and Equity Studies (CHESS), Karolinska Institute and Stockholm UniversityStockholm, Sweden; ^5^Department of Clinical Neuroscience, Karolinska InstituteSolna, Sweden

**Keywords:** catatonia, migration, culture-bound syndrom, pervasive refusal, psychogenic, apathy, hopelessness, predictive coding

## Abstract

Resignation syndrome (RS) designates a long-standing disorder predominately affecting psychologically traumatized children and adolescents in the midst of a strenuous and lengthy migration process. Typically a depressive onset is followed by gradual withdrawal progressing via stupor into a state that prompts tube feeding and is characterized by failure to respond even to painful stimuli. The patient is seemingly unconscious. Recovery ensues within months to years and is claimed to be dependent on the restoration of hope to the family. Descriptions of disorders resembling RS can be found in the literature and the condition is unlikely novel. Nevertheless, the magnitude and geographical distribution stand out. Several hundred cases have been reported exclusively in Sweden in the past decade prompting the Swedish National Board of Health and Welfare to recognize RS as a separate diagnostic entity. The currently prevailing stress hypothesis fails to account for the regional distribution and contributes little to treatment. Consequently, a re-evaluation of diagnostics and treatment is required. Psychogenic catatonia is proposed to supply the best fit with the clinical presentation. Treatment response, altered brain metabolism or preserved awareness would support this hypothesis. Epidemiological data suggests culture-bound beliefs and expectations to generate and direct symptom expression and we argue that culture-bound psychogenesis can accommodate the endemic distribution. Last, we review recent models of predictive coding indicating how expectation processes are crucially involved in the placebo and nocebo effect, delusions and conversion disorders. Building on this theoretical framework we propose a neurobiological model of RS in which the impact of overwhelming negative expectations are directly causative of the down-regulation of higher order and lower order behavioral systems in particularly vulnerable individuals.

## Introduction

In Sweden, apathy has been the colloquial term for a condition characterized by global and severe loss of function affecting children and adolescents seeking asylum or undergoing migration process. Typically prodromal anxiousness and depressive symptoms, in particular lethargy, progresses into stupor and finally complete lack of any response behavior even to painful stimulus. At this stage patients are seemingly unconscious and tube feeding life sustaining. After months to years remission ensues with gradual return to what appears to be normal function.

From January 1st 2003 to April 31st 2005, 424 cases were reported (Hessle and Ahmadi, 2006) and out of the 6547 asylum applications submitted for children (0–17 years) in Sweden in 2004 (Von Folsach and Montgomery, 2006), 2.8% were thus diagnosed. No cases reported from other countries, the phenomenon appears unique to Sweden.

The nature and prevalence of the condition has been subject to intense public debate. Malingering or Munchausen syndrome by proxy has been proposed. Opposing views have labelled these hypotheses xenophobic and instead suggested the migratory process, purportedly unpredictable and long, to precipitate the putatively stress-induced condition affecting traumatized individuals. An asserted “questioning attitude”, in particular within the health care system, it has been claimed, may constitute a “perpetuating retraumatization possibly explaining the endemic” distribution (Bodegård, 2014).

An official inquiry (Hessle and Ahmadi, 2006) and an expert committee (Rydelius, 2006) have both proposed multifactorial explanatory models involving individual vulnerability, traumatization, migration, culturally conditioned reaction patterns and parental dysfunction or pathological adaption to a caregiver’s expectations to interplay in pathogenesis. Severe depression or conversion/dissociation disorder has been suggested (Rydelius, 2006) and malingering or factitious disorder remain unsupported (Aronsson et al., 2009).

January 1st 2014, the Swedish National Board of Health and Welfare recognized the novel diagnostic entity *resignation syndrome* (RS; Socialstyrelsen, 2013). Implying a psychological etiology, its appropriateness remains to be demonstrated. In this presentation, the term will be used; however, should be interpreted free from theory.

As of today, diagnostic criteria are undetermined, pathogenesis uncertain and the regional distribution unexplained. New cases are presenting, 22 in the Stockholm area in 2014 (Schiller, 2015, Personal Communication), and effective treatment is lacking.

In this article we address three questions in relation to RS: What is it? Why is it locally distributed? And how can it arise? First we summarize and analyze the literature on RS. Then we argue: (1) that RS should be perceived as catatonia, a hypothesis readily testable by either neuroimaging or evaluation of treatment response; (2) that culturally transmitted and sanctioned beliefs may, through psychogenesis, account for the regional distribution; and (3) on a mechanistic level, that cultural and contextual influence may fundamentally change expectations and priors on the bodily functions inducing failure to activate both higher order and lower order behaviors in vulnerable individuals. To support this claim we frame it in a predictive coding context that has been suggested to be causally involved in placebo and nocebo effects, delusions and conversion disorders.

## Background

### Clinical Observations and Treatment

In a material of 23 patients, Bodegård (2005b) described the typical patient as “totally passive, immobile, lacks tonus, withdrawn, mute, unable to eat and drink, incontinent and not reacting to physical stimuli or pain”. He further noted that “[p]eriods of panicky refusal and/or anxiety can proceed or intervene with the stuporous state” and that “[s]econdary symptoms may appear, such as tachycardia, rise in temperature, weight gain, oedema, profuse sweating, reactivation (?) of latent viral infection, skin ulcers and muscular atrophy” (Bodegård, 2005b). Later reports and current observations find less evidence of “panicky refusal” and “secondary symptoms”. The persisting impression is that of symptoms progressing on a continuum from introversion and lethargy to stupor, lack of response and seeming unconsciousness.

Typically, non-negotiable symptoms, such as inability to ingest, elicit contact with the health care system. Sometimes a possible trigger incident, such as a negative asylum decision, can be identified. Patients may be admitted after a few days marked by rapid deterioration and stupor. On other occasions a more gradual presentation of anxiety, dysphoria, sleeping disturbances, social withdrawal and other symptoms are subsequently supplemented by mutism, failure to participate in activities such as school and play, failure to communicate altogether, and finally, to initiate motor activity and respond to stimulus leaving the patient in a supine position seemingly unconscious and generally with eyes closed (for clinical characteristics, see Box 1). At this stage, RS prompts tube feeding and full ADL-support (Bodegård, 2005b; Aronsson et al., 2009; Ascher and Hjern, 2013; Forslund and Johansson, 2013).

Box 1 **Clinical characteristics of RS.**

**Prodromal**

Anxiety

Dysphoria

Sleeping disturbances

Social withdrawal

**Deterioration**

Mutism

Failure to participate in activities

Failure in non verbal communication

**Fully developed**

Stupor

Unresponsiveness/negativism

Immobility

Incontinence

No reaction to pain stimulus including reflex

Dependent on tube-feeding and full ADL assistance

Tachycardia

Elevated temperature

Occasional profuse sweating

Occasional hyperventilation

Muscular atrophy

Periods of excitability, anxiety, “refusal”

Generally normal routine neurologic examination

Hypotonicity (sometimes coded as flaccid paralysis)

Reflexes generally responding but weak

Eyes open or closed not permitting passive opening

Pupils reactive to light and occasionally responsive to threat

Eyes divert from examiner and appear unseeing

Jerks

Indications of preserved awareness

Electroencephalogram (EEG), CT of skull and brain, laboratory sampling including toxicology all unimpressive

**Remission**

Generally in an ordered pattern (hand squeeze or eye opening without eye contact; eye contact; nodding and active partaking in feeding; gross motor skills; fine motor skills; verbal communication)

Indications of varying degrees of amnesia

Full recovery without remaining symptoms or deficits

Based on own experience and reports (Bodegård, 2005b; Aronsson et al., 2009; Ascher and Hjern, 2013; Forslund and Johansson, 2013).

Routine work-up includes toxic screening and anamnestic interviewing via interpreter. Neuroradiology, neurophysiological examinations and lumbar puncture are considered optional (Socialstyrelsen, 2013). Electroencephalogram (EEG) and computed tomography of the skull have generally been unimpressive as well as laboratory screenings (Bodegård, 2004; Aronsson et al., 2009; Forslund and Johansson, 2013). Magnetic resonance tomography (MRT) is recommended (Rydelius, 2006) however, rarely performed.

Once stabilized, somatic illness excluded and the parent(s) comfortable administering tube feeding, the patient is discharged and subsequent treatment given in a home setting with regular ambulatory visits to the clinic. In previous years long-term hospitalization was common and there is still a lacking consensus regarding level of care (Lindberg and Sundelin, 2005). Although Bodegård (2006) finds support for hospitalization in one report out-patient care aiming for family involvement is presently the preferred model.

Apart from life-sustaining tube feeding, treatment amounts to promoting and maintaining a secure and hopeful environment, encouraging a sense of coherence. Several authors stress the importance of a permanent residency permit (PRP; Lindberg and Sundelin, 2005; Ascher and Gustavsson, 2008) although a permit in itself neither is sufficient for remission nor precludes debut (Bodegård, 2006). Alongside care given by the family, nurses, psychologists, physiotherapists and occupational therapists are responsible for day-to-day care. A pediatrician and a child and adolescent psychiatrist are involved at regular intervals. One center employed individualized sense stimulation (Forslund and Johansson, 2013). No reports of successful pharmacological treatment, including antidepressants, or attempted electro-convulsive therapy (ECT) exist.

The duration of tube-feeding ranges from months to years. In one study (*n* = 29) the mean duration was 10, 6 months (1.1–24.5; Aronsson et al., 2009) and in another, inpatient study (*n* = 5), 27 weeks (10–60; Forslund and Johansson, 2013). Bodegård (2006) (*n* = 25) reports duration of tube feeding to covariate, in particular, with level of care (in favor of hospitalization); however, also with time elapsed between hospitalization and PRP being granted.

One study (*n* = 29) described remission following a similarly ordered pattern in 22 patients; hand squeeze or eye opening without eye contact was followed by eye contact, nodding and active partaking in feeding, return of gross motor skills, return of fine motor skills and verbal communication (Aronsson et al., 2009).

One survey (*n* = 5) report full recovery 1–8 years after discharge (Forslund and Johansson, 2013). At present, in relation to available data, the generally held assumption of “full recovery” remains poorly supported.

### Epidemiology

The patient group as hitherto described comprises of children and adolescents 7–19 years of age (mean 14.3) with 2:3 female predominance (Bodegård, 2004; Aronsson et al., 2009; Söndergaard et al., 2012; Forslund and Johansson, 2013). All described cases are refugees often belonging to a political or ethnic minority (Bodegård, 2005a, 2014; Forslund and Johansson, 2013). A disproportionally large share of patients originate from former Soviet republics or former Yugoslavia (Von Folsach and Montgomery, 2006) but cases from Bangladesh and Africa have been reported (Lindberg and Sundelin, 2005). The Uighur ethnic group is reported to be overrepresented among those affected (Rydelius, 2006). Traumatization in terms of physical abuse, harassment or by witnessing violence and abuse in the close family, is prevalent in half to all affected individuals (Bodegård, 2005b; Godani et al., 2008; Aronsson et al., 2009; Forslund and Johansson, 2013). Purported pre-morbid personality traits such as conscientiousness and high achieving have been reported (Forslund and Johansson, 2013).

To our knowledge, no cases have been established outside of Sweden. An accurate estimate of the total number of cases is challenging due to varying quality in reported materials. In 2002, 65 cases were reported and in 2004, 130. A national effort in 2003–2005 recognized 424 cases. In 2006 a temporary refugee amnesty easing asylum approval was enforced and later considered to contribute to a subsequent decrease (Socialstyrelsen, 2013). In 2005–2007, 70 patients were included in the rehabilitation programme in the Stockholm area alone (Aronsson et al., 2009), in 2013, 15 and in 2014, 22 (Schiller, 2015, Personal Communication). The prevalence as stated by the national effort 2003–2005 probably represents an exaggeration (Billing, 2014, Personal Communication). In an official inquiry from 2006 extensive interviewing, register inventories and field studies conducted in relevant countries and regions yielded no information of reaction patterns, nor of circumstances, such as cultural peculiarities, that could account for the phenomenon (Hessle and Ahmadi, 2006).

### Apathy

In relation to the epidemiological data it is not entirely unreasonable to at least speculate in a novel pathogenic entity. However, historic accounts of similar symptom panoramas exist arguably precluding this interpretation.

In 1913, Jaspers characterized apathy: “[t]his is the term given to absence of feeling” where “there is no incentive to act” manifested “objectively in the patient not taking food, in a passive indifference to being hurt, burnt, etc. The patient would die if we did not keep him alive with feeding and nursing care” (Jaspers, 1913/1993; see Box 2).

Box 2 **“Apathy” according to Jaspers (1913/1993).**

“This is the term given to absence of feeling. If this absence is complete, as can happen in acute psychoses, the patient is fully conscious and oriented, sees, hears, observes and remembers, but he lets everything pass him by with the same total indifference; happiness, pleasure, something positive in which he is involved, danger, sorrow, annihilation are all the same. He remains “dead with wakeful eyes”. In this condition there is no incentive to act; apathy brings about aboulia. It seems as if that one aspect of psychic life we call object-awareness has become isolated; there is only the mere grasp of reason on the world as an object. We can compare it to a photographic plate. Reason can portray its environment but cannot appreciate it. This absence of feeling shows itself objectively in the patient not taking food, in a passive indifference to being hurt, burnt, etc. The patient would die if we did not keep him alive with feeding and nursing care. The apathy of these acute states must be distinguished from the dullness of certain abnormal personalities who are constantly at the mercy of innumerable feelings, only crude in quality.”

(Jaspers, 1913/1993)

Children exhibiting lethargy and apathy with resemblance to depressive stupor or catatonia in connexion to traumatic events (Annell, 1958) and reaction patterns in catastrophes and war involving reduced contact and “apathic introversion” along with other symptoms interpreted to be psychosomatic (Otto, 1982), have been described.

Numerous phenomena resembling RS have been reported by physicians and anthropologists across contexts, cultures and time periods suggesting a common psychosomatic mechanism (Kihlbom, 2013). Acute as well as prolonged death ensuing real or magical threat of death is known from cultures on most continents (see e.g., Lester, 2009). “Epidemics” of dying in war and captivity where no hope remains has been described (Kihlbom, 2013). Nostalgia has been examined in relation to deterioration, apathy and dying (Johannisson, 2001). The concentration camp term “muselmann” denoted those void of all hope exhibiting resignation behavior (Kertész, 1998) claimed to sustain for weeks without nutrition in a state of “archaic autohypnosis” (Kihlbom, 2013). Unexpected and unexplainable sudden death following cancer diagnosis has been termed “self-willed death” (Milton, 1973). Sudden nocturnal death in Hmong immigrants in the USA (Adler, 1994) is hypothesized to result from sleep paralysis-type panic attacks involving punishment by spiritual encounter inspired by folk tales.

Resignation, apathy and eventually death in response to severe unavoidable threat is a consistent finding throughout history and across cultures.

### Diagnostic Conceptualizations

A wide range of diagnostic alternatives have been considered; various neurological disorders, anorexia nervosa, selective mutism, school refusal, social phobia, other anxiety states, states of conversion and dissociation, chronic fatigue syndrome, depression, catatonic states, and malingering. Among these none, according to Bodegård (2005a), fully exhaust the clinical picture including presentation, course and recovery. He therefore coined the term *Depressive Devitalization* (DD) only to later argue the condition, in its most severe form, to be identical to *Pervasive Refusal Syndrome* (PRS; Bodegård, 2005b) as introduced by Lask et al. (1991) and designating a child’s “dramatic social withdrawal and determined refusal to walk, talk, eat, drink, or care for themselves in any way”.

The similarities and differences between DD and PRS have been discussed (Von Folsach and Montgomery, 2006); PRS involves active refusal, DD in all its forms does not, and further, PRS does not manifest “flaccid paralysis and generalized sensory loss”, DD does. Accordingly, DD and PRS have been suggested to be subgroups of “the same refusal syndrome” (Von Folsach and Montgomery, 2006).

In a re-conceptualization of PRS, yet another term—*Pervasive Arousal-Withdrawal Syndrome* (PAWS)—was introduced together with an hypothesis of hyper-arousal in the sympathetic and parasympathetic autonomic nervous systems resulting in a “deadlock” manifesting itself in refusal, on this account re-conceptualized as a combination of “extreme anxiety avoidance” and “behavioral paralysis” mirroring the autonomic responses respectively. The authors predict high energy consumption as well as activity shifts in amygdala and insula to be present (Nunn et al., 2014). Interestingly, indirect calorimetry demonstrated energy expenditure below the requirement of basal metabolism in two patients suggesting an equivalent of hibernation (Jeppsson, 2013).

In contrast to the novel diagnostic entities such as DD, PRS and PAWS stand accounts relying on established diagnoses.

Several authors discuss stress-induced conditions such as *posttraumatic stress disorder* (PTSD) yet refrain, due to lack of diagnostic fit, from adopting these (Lindberg and Sundelin, 2005; Söndergaard et al., 2012; Bodegård, 2014).

An expert committee (Rydelius, 2006) identified severe depression or conversion/dissociation disorder to be the best diagnostic alternatives. Engström (2013), a member of the committee, argued traditional diagnostic entities sufficient in the majority of cases. He recognized RS as *severe major depressive disorder with psychotic features specified as catatonic* (DSM-IV 296.24), or in the ICD-10 taxonomy; as a *severe depressive episode with psychotic symptoms, in particular stupor* (F32.3).

January 1st 2014 the Swedish National Board of Health and Welfare, for epidemiological purposes, recognized *RS* (*uppgivenhetssyndrom*, ICD-10 F32.3A) and the specifier *problem adhering to status as refugee and asylum seeking* (Z65.8A). From a diagnostic viewpoint the introduction has been argued unnecessary (Engström, 2013). RS classified among the depressive entities (F32–33) should be interpreted as pragmatic solution to controversies regarding the nature of the phenomenon (Socialstyrelsen, 2013). Diagnostic criteria remain undetermined.

### Etiological Conceptualizations

An expert committee suggested six etiological conceptualizations (Rydelius, 2006). These included: (1) the *medical model of disorder* according to which a disorder affects vulnerable individuals under certain circumstances; (2) the *family model* stressing family psychology system theory; (3) the *psychological model* emphasizing effects of uncontrollability; (4) the *political model* identifying political decisions governing the asylum process; (5) the *cultural model* proposing the symptoms to instantiate a phenomenon belonging to either the patients’ cultural, religious or existential descent or to that of the country to which they migrate. Implicit in the cultural model lays the notion of secondary illness gain; and (6) according to the *intended model* an intentional decision made by the family or by the child explains the symptoms.

#### A Stress Hypothesis

Several authors endorse a stress hypothesis arguing a sustained stress response to be, if not the explanation, at least a contributing factor in pathogenesis (Lindberg and Sundelin, 2005; Söndergaard et al., 2012; Bodegård, 2014). Hypothetically, a sufficient and sustained “discrepancy between what is expected and what really exists” (Ursin and Eriksen, 2010) eliciting a stress response could precipitate debut in individuals predisposed by genetic, comorbid (depression, anxiety, neuropsychiatric disorders), premorbid (personality traits or adverse life events) or other unknown factors. Early symptoms accord with a stress induced condition (Lindberg and Sundelin, 2005) and altered autonomic function (tachycardia and rise in temperature) may be interpreted in analogy. The impact of a PRP on remission is taken to support the stress hypothesis and obtaining it is therefore considered an essential element in treatment (Lindberg and Sundelin, 2005).

Trauma and stressful events interplay with coping. This conjunction in turn impact on risk and resilience with regards to psychopathology (Ursin and Eriksen, 2010). Relatedly, one RS report (*n* = 29) surveyed predisposing, precipitating and perpetuating factors (Godani et al., 2008). In the neonatal period, 15 children exhibited predisposing factors associated with attachment (preterm birth, obstetric or neonatal complications, malformations, severe infection, congenital hip dysplasia etcetera). In the toddler period 25 had exhibited behavioral anomalies or had been subject to stressors (migration, loss of primary carer, severe illness, starvation, war, threat, death in family etcetera). Only a few individuals failed to demonstrate predisposing factors altogether. Putatively precipitating factors of either having witnessed or been subject to traumatization by threat, violence, rape or witnessing death, were demonstrated in all but one child. Indications of perpetuating factors, such as insufficient maternal care and ability to supply security, including previous maternal psychiatric illness and traumatization by assault, rape, murder of relative etcetera were, taken together, present in the majority of cases. The fathers’ contribution could not be studied due to insufficient data. Findings support predisposing and perpetuating factors being of considerable importance. Traumatization of mothers and children correlate inversely with time spent in Sweden prior to debut and directly with length of tube-feeding dependency (Godani et al., 2008). The material was biased towards advanced cases, and controls were lacking altogether.

In concordance with the stress hypothesis diminished diurnal variation of cortisol measured in saliva has been demonstrated, however in a small sample (*n* = 4; Godani et al., 2008). In another study (*n* = 11), patterns of endogenous steroids imply negative association of concentrations of cortisol and cortisone, and positive association with pregnenolone, 17-hydroxypregnenolone and dehydroepiandrosterone (DHEA) with severity of symptoms and the time of recovery (Söndergaard et al., 2012). No statistically significant difference in cortisol levels at entry and after recovery was shown. Elevated levels of DHEA and pregnenolone was demonstrated and suggested to support a *neurosteroid hypothesis of stress* (Söndergaard et al., 2012).

The reported overrepresentation within the Uighur ethnic group (Rydelius, 2006), it may be hypothesized, could result from a predisposing genetic or epigenetic factor. However, no cases of RS or similar phenomena were confirmed in the regions from which the Uighurs migrate (Hessle and Ahmadi, 2006).

The stress hypothesis suggests the condition to be present in comparable populations and in particular other refugee populations. To our knowledge no such reports exist. Personal communication with the child and adolescent psychiatrist Dr Abdulbaghi Ahmad, founding director of Metin Health House, a child mental health center in Duhok, Kurdistan, reveals no cases in the Duhok refugee camps accommodating approximately 100,000 people of Syrian decent and more than 600,000 internally displaced people from Iraq, among which about 28% are 5–14 years of age. Dr Ahmad, with expertise in childhood trauma, from Sweden and Kurdistan (Ahmad, 2008; Ahmad et al., 2008), reports various stress-induced conditions in the camps but none resembling RS. To account for the regional distribution, the stress hypothesis would need an auxiliary hypothesis.

#### A Psychodynamic Interpretation

A model implicating the mother’s predicament as the driving force behind RS has been proposed (Bodegård, 2005a). Inspired by the hypothesized mechanisms underpinning PRS (Nunn and Thompson, 1996), Bodegård suggests a psychodynamic interpretation.

The majority of mothers in Bodegård’s material had been subject to physical and or sexual abuse (Bodegård, 2005a; Godani et al., 2008) and were described as severely traumatized. Their attitude was signified by a lack of trust, rejection of medical information excluding physical illness as causing the condition and resistance to rehabilitation and treatment on the child’s behalf. On Bodegård’s interpretation, this attitude and the corresponding behavior may be perceived as parts of a coping strategy by which the mother’s traumatized depressed situation and need for consolation is projected from herself and onto her child. She creates a “delusive fantasy of the child as dying” and the child acting to maintain the right to be its mother’s child, a folie à deux implicating the idea of a serious illness is staged. By “lethal mothering” the mother unconsciously creates and maintains an alternative reality in which she finds meaning in caring for a child imagined as dying in turn affecting the child and promoting debut and progression of the disorder.

The situation can be related to a Munchausen by proxy scenario in which the mother’s delusion, aimed at concealing her own desperate situation by projecting it to the imagined disorder of the child, distorts not only her reality but also that of the child which in the process is abandoned and forced into adapting the role of dying or, “devitalized”. From the child’s perspective the prospect of rejection by the mother is more frightening (on a subconscious level) than adopting the delusion which protects not only from rejection itself but also from the emotional trauma of failing mothering. In relation to this interplay the child’s deterioration, withdrawal, stupor and finally, full blown DD, may be conceptualized on a psychodynamic interpretation according to Bodegård (2005a).

Other authors fail to report evidence of inadequate mothering or disadvantageous maternal coping strategies. The hypothesis would suggest the phenomenon to be present in comparable populations. Such reports have failed to reach the research community.

Interestingly, a notion of expectancy as a contributor in pathogenesis is invoked. The staging of the child as dying and it acting accordingly, would serve to illustrate how a propagated set of beliefs may govern reaction patterns. Also, Bodegård’s proposal involves a family system perspective attractive in relation to the observation that, to our knowledge, RS in unaccompanied minors have not been observed.

## Hypotheses

In relation to the nature and regional distribution of RS neither of the two examined hypotheses—the stress hypothesis and the psychodynamic hypothesis—are sufficient. Both, although possibly of importance, fail to account for the regional distribution and predict the disorder to be present in populations where it is not. We now proceed to argue that catatonia satisfyingly fits the clinical characteristics of RS and that the regional distribution can be explained by invoking a notion of culture-bound psychogenesis.

### RS is Catatonia

Rather than a lack of awareness, RS is characterized by an inability to initiate motor response, a finding also present in catatonia and conversion disorder. On the basis of substantial clinical overlap, we argue that RS should be perceived as catatonia. As catatonia promptly responds to a test dose of lorazepam (or equivalent) and is validated by positive treatment effect with benzodiazepines and/or ECT (Fink and Taylor, 2003); and as neuroimaging may indicate altered brain activity in catatonia (De Tiége et al., 2003), as well as preserved awareness (Vanhaudenhuyse et al., 2010) the hypothesis is testable.

#### Arousal and Awareness in RS

At its most advanced stage RS patients appear unconscious. The eyes of the patients are generally closed and remain so despite stimulation. If passively opened eyes sometimes diverge away from the examiner. Further, patients exhibit (what has been interpreted as) flaccid paralysis or hypotonicity and complete lack of pain response (sternal rub, supraorbital pressure, nail-bed pressure) as well as reaction to extraction or insertion of nasogastric tube. We are unaware of caloric testing having been performed in order to determine physiological nystagmus indicative of wakefulness. An “Amytal interview”^1^ (Iserson, 1980; Posner et al., 2007) or a benzodiazepine challenge^2^ (Fink and Taylor, 2003) has to our knowledge not been exploited in order to reveal a psychogenic state. Interestingly, however, Bodegård (2005a) reports of two patients temporarily normalizing following midazolam administration prior to insertion of a nasogastric tube. Nevertheless, a condition lacking both arousal and awareness is the general impression when examining RS patients.

The general impression needs however be questioned. Sleep-wake cycles are indicated by hypnagogic jerks and confirmed by EEG-recordings (Bodegård, 2005a). Language acquisition in the seemingly unaware state, tear excretion in otherwise detached faces, self-report of inclination to console parents in despair as well as of blurred visions including “fairies” all testify to preserved awareness (Engström, 2013). Bodegård claims full awareness (*n* = 5) during the course of the disorder and negates amnesia (Bodegård, 2005a). Another study reports varying degrees of amnesia (Forslund and Johansson, 2013).

According to these reports RS exhibits a combination of inability to respond to any stimulation and maintained, perhaps fluctuating, awareness, as well as preserved arousal. Neither arousal nor awareness thus appear impaired to an extent explaining the lack of response to painful stimulus. Accepting this line of argument, the inability to initiate motor activity would have to account for unresponsiveness, which indeed has been proposed (Engström, 2013). On this interpretation, RS is consistent with psychogenic unresponsiveness possibly on the basis of catatonia or conversion disorder both known to generate motor symptoms of either inhibitory or excitatory nature (Posner et al., 2007).

#### Catatonia

Recently a considerable shift has occurred in the conception of catatonia (Tandon et al., 2013). For a long period considered a sub group within schizophrenia, in DSM-IV catatonia was partly separated from this hierarchy by the addition of a new class; *Catatonia secondary to medical condition*. Successful treatment in catatonia exhibiting little or no effect in schizophrenia, and catatonia occurring in relation to other psychiatric as well as somatic disorders motivated the separation, which in DSM-5 was finalized by the deletion of *schizophrenia, catatonic type* altogether. Currently catatonia is conceived of as a neuropsychiatric syndrome associated with systemic illness (Fink, 2013; Fink et al., 2015).

In DSM-5, catatonia is defined as the presence of three or more symptoms out of a list of twelve (Table 1). Among these, stupor, mutism and negativism are all general finding in RS (Box 1). Diagnostic criteria apply regardless of age. Nevertheless, pediatric catatonia has been suggested to consist of three cardinal symptoms; immobility, mutism and withdrawal or refusal to ingest (Takaoka and Takata, 2003). Depending on clinical presentation, either the specifier *with catatonia* together with major depressive disorder, or, the separate entity *catatonic disorder NOS* (*not otherwise specified*; Tandon et al., 2013) would be applicable to RS. From a phenomenological perspective, applying these diagnostic labels should meet no resistance.

**Table 1:** **Catatonia in DSM-5**.

Catatonia is defined as the presence of three or more of the following:	
1. Catalepsy	Passive induction of a posture held against gravity
2. Waxy flexibility	Slight and even resistance to positioning by examiner
3. Stupor	No psychomotor activity; not actively relating to environment
4. Agitation	(Not influenced by external stimuli)
5. Mutism	No, or very little, verbal response (not applicable if there is an established aphasia)
6. Negativism	Opposing or not responding to instructions or external stimuli
7. Posturing	Spontaneous and active maintenance of a posture against gravity
8. Mannerisms	Odd caricature of normal actions
9. Stereotypies	Repetitive, abnormally frequent, non-goal directed movements
10. Grimacing	
11. Echolalia	Mimicking another’s speech
12. Echopraxia	Mimicking another’s movements
	American Psychiatric Association (2013)

Posner et al. (2007) characterize catatonic stupor (as opposed to the excited form): the patient’s eyes are usually open apparently unseeing, or sometimes, tightly closed resisting passive opening. Skin is pale and acne or oily skin common. Pulse is rapid (90–120) and temperature often elevated (1.0–1.5°C). Spontaneous movement is rare and unawareness the impression. Pupils are dilated and reactive to light, alternating anisochoria is common and opticokinetic response present, however, patients’ may fail to blink to visual threat. Doll’s eye test is negative and caloric testing produces normal ocular nystagmus. Increased salivation is sometimes noted. Incontinence may be present. Urinary retention may require catheterization. Extremities are relaxed or rigid resisting passive movements. Catalepsy (waxy muscular/postural rigidity and reduced responsiveness) is present in 30%. Choreiform jerks of the extremities and grimaces are common. Reflexes are normal. Consciousness is preserved although the appearance is the opposite. On recovering, the patient is often, but not always, able to recall events that occurred during illness. Normal neurological examination and self-reports after recovery attest preserved consciousness.

Further, inability to speak despite urge to do so, as reported in an RS patient (Engström, 2013), has been reported in Catatonia (Fink, 2013) and after remission, catatonic patients recover fully which appears to be the case also in RS patients (Forslund and Johansson, 2013) although this finding need to be confirmed. “Panicky refusal” (Bodegård, 2005b) may be interpreted as agitation, a common finding in the exited form of Catatonia (Fink and Taylor, 2003).

Reviewing the symptoms of RS and catatonia, (Box 1, Table 1) resemblance is undeniable. Clinical characteristics of RS match the diagnostic criteria of catatonia. Dhossche et al. (2012) argue pediatric catatonia to be the genuine diagnosis in both RS and PRS and find evidence of deprivation, abuse and trauma to precipitate catatonia in children and adolescents. Shorter (2012) contests the prevailing belief that pediatric catatonia is a rare disorder; other diagnostic labels have obscured the condition (Table 2), which, prior to Kahlbaum coining the term in 1874, was only natural. An extensive review of catatonia in all age groups supports Shorter’s analysis (Fink, 2013). Cohen et al. (1999), based on a literature review, report 42 cases of adolescent catatonia among which 19 were associated with mood disorder. Posner et al. (2007) suggest catatonic stupor to be rare due to effective treatment. This is of course only applicable if the condition is recognized and treated.

**Table 2:** **Diagnostic labels that have historically obscured Catatonia as an independent disease according to Shorter (2012)**.

Pre 1850s	Stupor
	Catalepsy
	Stupidité
	Death spells
1869	Neurasthenia
	Hysteria (dissociated from Catatonia in 1920s)
1871	Hebephrenia
1874	Catatonia
1899	Dementia praecox
1903	Psychasthenia
1908	Schizophrenia
1920s	Encephalitis lethargica
1934	“Brain stem” changes (precursor to ADHD)
1943	Autism
1991	Pervasive refusal syndrome
2007	Anti-NMDA receptor encephalitis

#### Demonstrating Catatonia

In acute catatonia, treatment effect verifies the diagnosis: prompt response to a benzodiazepine challenge implies catatonia and treatment effect with benzodiazepines and/or ECT validates the diagnosis (Fink and Taylor, 2003). As already noted, Bodegård (2005a) observed two patients temporarily normalizing in response to midazolam. In acute catatonia, 60–90% responds to lorazepam (Northoff, 2002; Fink and Taylor, 2003). Chronic cases may fail to respond (Northoff, 2002). Amantadine may have effect in these cases and ECT, considered the most potent alternative, exhibits effect in 80–100% of all cases (Luchini et al., 2015).

Pediatric catatonia is typically treated with benzodiazepines and ECT (Dhossche et al., 2009; Weiss et al., 2012; Wachtel et al., 2013). In a pediatric population ECT is considered effective and safe. There are no studies indicating deleterious side-effect and the fear of inflicting damage to the developing brain finds no support (Wachtel et al., 2011). Interestingly, the first five patients receiving treatment with convulsive therapy in 1934 were stuporous and had required tube-feeding for several months; repeated intramuscular injection of camphor precipitating seziures were effective in all patients (Luchini et al., 2015).

Posner et al. (2007) conceive of catatonia as psychogenic unresponsiveness (which is not to say it is imagined). In the clinical context, psychologically induced neurological symptoms usually exhibit normal EEG and MRI findings (Posner et al., 2007); however, using positron emission tomography (PET) technology, regional metabolic abnormalities have been demonstrated including reduced metabolism in the prefrontal cortex (the anterior cingulate, the medial prefrontal and dorsolateral cortices) in a 14 year old girl diagnosed with akinetic catatonia in the context of Bipolar type 1 disorder (De Tiége et al., 2003). Interestingly, anterior cingulate cortex (ACC) lesions are known to contribute to a range of behavioral disorders including akinetic mutism, diminished self-awareness, impaired motor initiation and reduced pain response (Devinsky et al., 1995). Posner et al. (2007) predict abnormal brain metabolism in *psychogenic coma*. Evaluation of prefrontal metabolism in RS patients is an attractive, so far unexplored, diagnostic alternative.

#### Demonstrating Awareness in RS

If RS is catatonia, consciousness should be preserved. In RS the general impression is that of a condition void of arousal as well as awareness, which by definition implies unconsciousness, yet indications of the opposite exist (Bodegård, 2005a; Engström, 2013). The unresponsive RS patient exhibiting only *that* behavior, bedside tests are inadequate. A similar situation faces the clinician examining patients suffering from *disorders of consciousness* (DOC) where residual awareness may be impossible to determine with traditional methods (Giacino et al., 2009). However, through analysis of brain resting state activity a further means of discriminating between the unaware and aware patient has been made possible (Owen et al., 2006; Vanhaudenhuyse et al., 2010; Evers and Sigman, 2013).

Aberrant activity in the *default mode network* (DMN) has been demonstrated to correlate with a number of psychiatric and neurological disorders (Zhang and Raichle, 2010) as well as with physiological and induced variations in level of consciousness (Heine et al., 2012). Importantly, the level of consciousness in patients suffering from DOC have been described vis-à-vis activity in the DMN: functional as well as structural connectivity, established by exploiting fMRI BOLD-signal and diffusion tensor imaging (DTI) respectively, have been demonstrated to correlate with levels of consciousness thus discriminating between unaware and aware patients (Vanhaudenhuyse et al., 2010; Fernández-Espejo et al., 2012).

RS, like the DOC, may benefit from characterization by means of fMRI-BOLD resting state analysis. Undoubtedly different mechanisms generate DOC and RS. Nevertheless, the covariance between level of consciousness and DMN connectivity also in anesthesia (Ramani, 2015), hypnosis (Vanhaudenhuyse et al., 2014) and sleep (Horovitz et al., 2009) implies the DMN connectivity of interest in relation to level of consciousness regardless of cause. Resting state analysis could indicate to what extent RS patients are conscious and demonstration of preserved awareness would indirectly support RS being catatonia. It would also imply feasibility of neuro-technological communication (Owen et al., 2006; Evers and Sigman, 2013).

#### Interim Summary

Catatonia is from a phenomenological and clinical perspective an adequate label of what has been labelled RS. The reluctance to this attribution may be explained by unwillingness to ECT in children in Sweden (Shorter, 2012) and the up until recently prevailing view of catatonia as a sub group within schizophrenia. Catatonia prompts ECT or benzodiazepines. To our knowledge no RS patients have received such treatment. Residual hesitance may be overcome by at test dose of a benzodiazepine or by performing a PET examination to objectify suggested reduced prefrontal metabolism. Clinical observations implying preserved awareness may be evaluated further by resting state network analysis. The reconceptualization of catatonia invites to a re-evaluation of RS, more so now than ever, and its correspondence to catatonia.

### Culture-Bound Psychogenesis Explains the Regional Distribution of RS

Regardless of the relationship between catatonia and RS, the question remains how to explain the regional distribution of RS. Expressions of distress are constrained by brain function upon which beliefs and expectations impact. Evolving and transpiring within cultural contexts, beliefs and expectations readily serve as vehicles of *idioms of distress*. Culture-bound psychogenesis, it will be argued, may explain the regional distribution of RS. Genetic or environmental factors are conceivable in accounting for the regional distribution, nevertheless; such commendable analyses are beyond the scope of this article.

#### Clinical Traditions

Differences in diagnostics and treatment across countries conceivably supply explanations for the endemic distribution of RS. Provided the condition is promptly reversed, patients would not reach the prolonged stuporous state RS exhibits. This hypothesis predicts the incidences of catatonia—and/or other similar disorders—and RS to correspond.

In 2003–2005, the estimated annual incidence of RS was 2.8% in 0–17 year old asylum seekers. Catatonia incidence has been examined in two pediatric and adolescent psychiatric materials and found to be 0.6 and 5.5% respectively (Cohen et al., 1999; Thakur et al., 2003). The general incidence in the young population was estimated at 0.16% in Paris (Cohen et al., 1999). Estimated RS incidence is thus comparable to that of catatonia in psychiatric materials but does not correspond to that in a general material.

This discrepancy could reflect the vulnerability refugees as a group exhibit and the high incidence interpreted accordingly. Similar incidences would then be expected in comparable populations—in particular refugees populations—which, to our knowledge, remains to be surveyed in this respect. However, were the incidence of catatonia in young refugees in the vicinity of 2.8%, it would most likely have been reported, and; thus, differences in clinical practice are not likely to account for the regional distribution of RS. Possibly, however unlikely, other diagnostic entities could obscure RS in other refugee populations.

Billing (2014, Personal Communication) proposed too liberal diagnostic inclusion could explain the peak in incidence 2003–2005. However, this proposal does not explain the regional distribution *per se*. Instead, it illustrates the importance of perceiving a diagnosis as more than the label of a clinical entity. It invites the discussion of the diagnosis as a culturally influenced construct and an analysis of its application within a cultural context.

#### Culture-Bound

Yap (1962), in order to unify and retain traditional nosology, proposed the class “atypical culture-bound psychogenic psychoses” (later culture-bound syndromes) on recognizing the “pathoplastic influence” effected by culture to generate in “exotic psychoses”. Consequently, *Latah*, *Susto*, *Koro*, *Dhat* etcetera, were conceptualized as, and grouped among, the “reactive psychoses (psychogenic reactions)” (Yap, 1967). By *culture-bound* it was implied that “[w]ith respect to the psychogenic reactions, significant etiological factors are commonly to be found at the social and psychosocial level rather than the anatomical and biochemical” (Yap, 1967).

Although transcultural differences in psychiatry are controversial (Kleinman, 1987; Prince and Tcheng-Laroche, 1987; Keshavan, 2014; Ventriglio et al., 2015) they are evident; the incidence, symptoms, course and outcomes in schizophrenia (Myers, 2011); clinical presentation of depression and anxiety (Kirmayer, 2001), and; symptoms, self-perception, help-seeking behavior and treatment in relation to war trauma (Miller et al., 2009; Hinton and Lewis-Fernández, 2010; Shannon et al., 2015) vary across cultures. In recognition, all mental distress is, in DSM-5, considered culturally framed and populations expected to display culturally determined differences in communicating distress as well as in relation to explanations of causality, coping-methods and help-seeking behaviors (American Psychiatric Association, 2013). Consequently, culture-bound syndromes are recognized and grouped within the *cultural concepts of distress* defined as “ways cultural groups experience, understand, and communicate suffering, behavioral problems, or troubling thoughts and emotions” (American Psychiatric Association, 2013).

By *culture-bound* we recognize the impact exerted by socio-culturally transferable beliefs and expectations on an individual or population.

Many consider dualism an out-dated metaphysical basis for psychiatry (Shorter, 2006). In cognitive neuroscience the connexion between psychology, brain physiology and behavior is nevertheless indisputable and everyday life as well as clinical experience informs of the relevance of psychological processes to behavior. To demonstrate the impact of culture and context on symptom generation and presentation we draw on an account of psychogeninc illness (Shorter, 1992) exemplified by “La Grand Hystérie”, epidemic hysteria and suggestion. These phenomena are presented to illustrate the likelihood of RS being culture-bound.

#### “La Grande Hystérie”

Shorter, in an extensive analysis of the history of psychogenic illness, explores the relationship between physicians, patients and conceptions of disease throughout centuries (Shorter, 1992). In essence, he argues that cultural context–in particular diagnostic techniques, medical paradigms, familial expectation and social roles–influences what symptoms are legitimate and illegitimate by associating to them underlying organic disease for which the patient cannot be blamed, and; that unconsciously, in response to stress, trauma or suggestion, symptoms are assimilated from the “symptom pool” of legitimate symptoms and perceived as genuine indicators of an organic disorder or dysfunction by patients and physicians alike.

In treating patients with “hystero-epilepsy” at La Salpêtrière hospital in Paris, Jean-Martin Charcot developed a theory asserting that hysteria was an inherited, life-long, disease of the nervous system with sensory (headache, loss of sensation etcetera) and motor (tremor, paralysis etcetera) stigmata accompanied by reoccurring fits characterized by four phases presenting in a law-like manner: (1) the epileptiod period; (2) the “period of contortions and grande mouvements” during which the patients flung themselves about, crying and adopting improbable postures like “arc-de-cercles”; (3) the period of “impassioned poses” like prayer, crucifixion etcetera; and (4) a “terminal period” where anything could happen. Ovary tenderness at debut of fits was considered pathognomonic as well as hypnotisability.

Treatment—consisting of “metallotherapy” and hypnosis— and patient demonstrations, attended by students, visiting physicians, journalists and the general public, produced “a climate of suggestion” prompting patients to exhibit symptoms in accordance with the “laws of hysteria”. Scientific and journalistic reports paralleled the spread and increase of cases with predicted symptomatology. Eventually patients were referred from other continents.

On observing startle shock and suggestion by hypnosis precipitating the symptoms, Charcot later came to recognize psychological factors as possible inducers of hysteria. This shift Shorter interprets as the beginning of the end for “Charcot’s Hysteria”. No longer an organic disorder—and patients less prone to unconsciously select and present symptoms indicating a problem “merely in the head”—the incidence dropped. Also, Charcot’s successor, attributing the “epidemic” to iatrogenic suggestion, prohibited mention of hysteric symptoms in front of patients and ferociously challenged those exhibiting fits. Babinski, a student of Charcot’s—and the discoverer of a clinical procedure useful in distinguishing hysteric from organic paralysis—later characterized hysteria in La Salpêtrière as “any symptom that could be induced by suggestion [understood as medical or cultural] and abolished by persuasion [including hypnosis and psychotherapy]”.

“La Grand Hystérie” illustrates how psychogenic symptoms evolve over time, transpire epidemically and affect by suggestion. According to Shorter, the content of the symptom pool evolve constantly, through the continuous negotiation between physicians and patients immersed in cultural context, and is reflected in “pathoplasticity”, the changing psychogenic symptomatology. Current negotiations are affected in particular by media, and the contemporary expressions—controversially—include *chronic fatigue syndrome* and *environmental hypersensitivity* (Stewart, 1990; Shorter, 1992).

#### Epidemic Hysteria

*Epidemic hysteria* (Boss, 1997) of *mass sociogenic illness*—the rapid spread of unconsciously exhibited symptoms indicative of excitation, loss or alteration of neurological function without corresponding etiology in a cohesive group—exhibit contagious characteristics, and has been asserted, due to surface heterogeneity, to represent an under-appreciated, social problem (Bartholomew and Wessley, 2002).

Examples include regionally dispersed “dancing mania”—known as the St Vitus dance—reoccurring throughout the Middle Ages; motor hysteria outbreaks in nunneries or, more recently, boarding schools (reported from Malaysia during the 1980s), and; *mass hysteria*—often in poor working environment and related to mysterious odors (Boss, 1997). Continuous anxiety or stress in segregated highly controlled groups has been suggested to engender dissociation and hyper-suggestibility eliciting delusions reflecting the Zeitgeist, epidemic hysteria thus mirroring its time (Bartholomew and Wessley, 2002).

Recent reports include mass psychogenic illness (*n* = 170) attributed to toxic exposure at a high school (Jones et al., 2000); an outbreak of conversion disorder (*n* = 5) among Amish adolescent girls (Cassady et al., 2005), and; acute stridor (*n* = 12) in a female cohort of students in preparation for national exams (Powell et al., 2007).

#### Suggestion

Socio-cultural impact on individual psychogenic expressions has also been studied. Patients present symptoms in relation to social surroundings, iatrogenic suggestion (Fallik and Sigal, 1971) and following hypnosis (Halligan et al., 2000). Also, symptom attribution varies with “trendy diagnoses”: In 1985, most patients with environmental hypersensitivity disorder (*n* = 50), also attributed their problems to food and synthetics, in 1986 to Candida albicans, and in 1987 to chronic Epstein-Barr virus (Stewart, 1990). Contemporary fixed illness attributions have been suggested to align with media reports, and controversially, *chronic fatigue syndrome*, *myalgic encephalomyelitis* and *environmental hypersensitivity* are examples from our time (Shorter, 1992).

#### RS is Culture-Bound

Psychogenic symptom expression paralleling progression in medicine and culture (Shorter, 1992; North, 2015), discrete episodes of epidemic hysteria (Levy and Nail, 1993; Boss, 1997; Bartholomew and Wessley, 2002) and intra-individual presentation as well as progression of psychogenic illness attributions relating to trends (Stewart, 1990; Shorter, 1992) suggest culture-bound psychogenesis to be a robust and important phenomenon. The acknowledgment of transcultural differences (American Psychiatric Association, 2013), idioms of distress (Ventriglio et al., 2015) and varying psychogenic illness presentation supply indirect evidence culture-bound transfer of psychopathology and symptom induction by hypnosis (Halligan et al., 2000) provide direct evidence.

*Mass psychogenic illness*, traditionally *epidemic hysteria*, exhibit certain characteristics (Levy and Nail, 1993; Boss, 1997; Bartholomew and Wessley, 2002). Highly segregated groups where stress, control or obligations are evident and inescapable are predisposed and historically in particular religious settings are overrepresented. Female patients predominate. Patients below 20 years of age are overrepresented. Epidemics involve typical symptoms, including fatigue and unconsciousness, without demonstrable organic lesions. Relapse is common. “Compensational” issues have been reported of importance. Media reports are known to enable transmission of illness behavior.

RS mostly afflicts individuals of the same ethnic group, language community and previous, as well as present, cultural context (Rydelius, 2006) in which psychological and or physical trauma as well as stress are prevalent (Godani et al., 2008). Helplessness and hopelessness—equivalents of inescapability—are generally asserted (Bodegård, 2005a; Lindberg and Sundelin, 2005). Uighurs early trust children with high responsibility (Rydelius, 2006) something the predicaments of migration and asylum seeking may reinforce creating more of control and obligations. The male to female ratio is 2:3, mean age 14.3 years old and relapses have occurred. Symptoms imply a central nervous affliction, however, none have been demonstrated. A secondary illness gain may be assumed, as severe illness hypothetically generate in increased chances of asylum approval. On a different level the seemingly unconscious state may *per se* be perceived as a secondary illness gain offering relief. The estimated peak in RS cases was paralleled by extensive media reports, popularization and an infected debate—regarding in particular etiology, malingering and level of care—and involving, in a transparent way, also the medical profession (Hacking, 2010) supplying ample opportunities for the negotiation and transpiration of legitimate symptoms.

The RS endemic fails to demonstrate a clear index case (which there nevertheless may have been), an identifiable trigger event (although individual presentation sometimes is preceded by e.g., a negative asylum decision) and it is uncertain to which degree individual cases have been in contact prior to presentation. These factors are generally seen in epidemic hysteria (Boss, 1997).

Not described in other parts of the world and overrepresented in ethnic minorities from certain parts of the world, RS respects national borders and to some extent, ethnicity and/or language community. These peculiar circumstances are difficult to explain without reference to culture and context and we therefore assert RS to be culture-bound.

### Psychogenesis

In the previous section the notion of psychogenesis was inherent and served to transform culturally transpiring idioms of distress into generation of corresponding symptoms. Such neurological dysfunction in the absence of demonstrable organic lesion has been know to physicians since ancient times as *hysteria*. Grouped either among *conversion disorders* (DSM-5) or *dissociative disorders* (ICD-10), symptoms encompass loss, excitation or alteration of motor and sensory functions, including altered states of consciousness, sometimes in conjunction. Symptoms are genuine, sometimes disabling, and common–in one study functional and psychological symptoms were found to account for 16% of diagnosis in neurology units (Stone et al., 2010). Importantly, the symptoms are also involuntary, a fact not consistently recognized.

From Latin “hysterus”, hysteria originally implied an etiology involving dysfunction or displacement of the uterus. Charcot recognized suggestion or psychogenic shock to precipitate symptoms—treatable with hypnosis—and proposed abnormal or absent “mental imagery” to result in corresponding neurological dysfunctions (Shorter, 1992; Gelder, 2001). Janet, invoked traumatic narrowing of attention with subsequent *dissociation* and disintegration of mental processes creating unconscious yet processed mental realms (Gelder, 2001). Breuer and Freud (1956/1893) adopted this notion in their psychodynamic theory of *conversion* in which negative emotions ensuing “psychical trauma” were hypothesized to convert into symbolic physical symptoms resulting in *primary* and *secondary illness gain*. Invoking “a morbid condition of emotion, of idea and emotion, or of idea alone” in pathogenesis, Reynolds (1869) appreciated emotive as well as cognitive dysfunction.

The most commonly reported symptoms—*psychogenic non-epileptic seizures* (PNES), loss of consciousness and motor symptoms (Brown and Lewis-Fernández, 2011)—imitate organic disorders. Prevalence is increased following brain injury (Eames, 1992), prior to debut of, and parallel to, epilepsy (Devinsky et al., 2011), with depression, PTSD (Ballmaier and Schmidt, 2005), anxiety and borderline personality disorder (Brown and Lewis-Fernández, 2011). Although transculturally understudied (Brown and Lewis-Fernández, 2011), functional disorders have been claimed to vary little in incidence and semiology across cultures (Carota and Calabrese, 2014). Importantly, complex behavior, such as pseudo-labor, Genser syndrome, anorexia nervosa and catatonia, has been attributed to conversion (Jensen, 1984; Lyman, 2004; Jiménez Gómez and Quintero, 2012; Shah et al., 2012; Goldstein et al., 2013) implicating also higher order processes. Moreover, *de facto* organic findings in conversion disorder (Ballmaier and Schmidt, 2005; Vuilleumier, 2005, 2014; García-Campayo et al., 2009) indicate, contrary to the traditional conception, the possibility of a neurocognitive mechanism answering to symptom generation, and conversion disorder thus being a phenomenon, also, of the brain.

Reflecting the multitude of mechanisms and etiologies suggested, current DSM and ICD nosology is “widely regarded as unsatisfactory” (Gelder, 2001) in particular with regards to clinical overlap between conversion, dissociation and somatization (Brown and Lewis-Fernández, 2011; North, 2015), and mechanistic as well as etiological bias involving unconscious mental states and psychological stress or trauma, with undecided, little, or no empirical relation to symptoms (Roelofs and Spinhoven, 2007; Brown and Lewis-Fernández, 2011). Although the DSM-5 criterion involving identification of a specific psychological cause has been abandoned and *functional neurologic symptom disorder* (FNSD) introduced as an alternate term to conversion disorder (American Psychiatric Association, 2013), more extensive reclassification has been proposed (Brown et al., 2007; North, 2015).

In the previous section culturally determined expectations and beliefs were demonstrated of importance to symptom generation of culture-bound phenomena (Stewart, 1990; Shorter, 1992; Levy and Nail, 1993; Boss, 1997; Hinton and Lewis-Fernández, 2010; Medeiros De Bustos et al., 2014). Even so, a dogmatic psychological approach has been asserted “misguided and unhelpful” (Edwards and Bhatia, 2012) as psychological factors, particularly understood as trauma or internal conflict, not consistently are supported clinically or in epidemiological studies (Roelofs and Spinhoven, 2007; Brown and Lewis-Fernández, 2011). Moreover, inorganic genesis has been denied altogether (Slater, 1965) perhaps signaling dualism to some an out-dated metaphysical basis for psychiatry (Shorter, 2006). In cognitive neuroscience the connexion between psychology, brain physiology and behavior is nevertheless indisputable and everyday life as well as clinical experience informs of the relevance of psychological processes to behavior.

In general, the presupposition of physical symptoms occurring unattended by demonstrable organic findings, where there are strong evidence or presumptions that the symptoms are linked to psychological factors, seems to force an unwarranted and unfortunate mutually excluding, dichotomy creating a divide between neurological and psychological mechanisms. Either it is in the mind or, it is in the body. This starting point is infertile and so, denying *psychogenesis*—understood as implying psychological impact on symptom generation and precipitation—altogether, is equally absurd as is the opposite.

However, as the controversies regarding mechanisms and etiologies indicate fundamental difficulties in conceiving of the pathophysiology (Gelder, 2001; Roelofs and Spinhoven, 2007; Brown and Lewis-Fernández, 2011; North, 2015), an analysis of psychogenesis, relying on current nosology, is from the outset likely to perpetuate previous unhelpful conceptions. Ultimately, a model appreciating the impact of beliefs and expectations in directing and generating symptoms is the ambition. Consequently, although aspiring to neutrality, also *culture-bound psychogenesis* should be considered preliminary and any mechanistic analysis of symptom generation preferably be unbiased even in relation to, although not inconsistent with, psychological causation.

Relying on a framework of predictive coding, a mechanism answering to the protean nature of phenomena attributed to psychogenesis, we argue, may be attained. Importantly, such a mechanism permits also organic genesis of symptoms. Nevertheless, in relation to the present context—the notion of culture-bound serving to explain the regional distribution of RS—it should be emphasized that a description solely on the level of the brain is unlikely to be successful.

## A Predictive Coding Model

The effect of expectations on biological systems has been shown to be a decisive factor in both health and disease. The common denominator may be found in the models of predictive coding as a fundamental way the brain processes information. The general idea that the models of the world harnessed within the brain impact how we experience the world itself have been proposed more than a hundred years ago by Helmholtz (Helmholtz, 1866; Frith, 2007). Modern predictive coding theories suggest that Bayesian inference describe these processes (Friston, 2005; Frith, 2007).

Conceptually, such predictive coding hypotheses suggest that the brain constructs models of the world on different hierarchical levels. The models also act as expectations or predictors (priors) of the external and internal worlds. When a signal reaches the brain in a primary sensory region it will be compared with the priors, and if it does not match (in that the expectations are different as compared to the signal) it will produce an error signal. This error is proportional to the difference between prior and input, and will be propagated to the next hierarchical level where it is compared to priors on the intermediate levels. If these priors fail to explain the error signal it will continue its propagation to higher order hierarchies. The error signal may be used to change the priors or models of the world. However, the priors and the models may also change the way input is processed or perceived. Thus, a percept is determined both by the input and by the model. In Bayesian terms the models are conceptualized as *priors* and input as *observation* while the percept (thus dependent on both the prior and the observation) is often referred to as the *posterior*.

Research on placebo and nocebo treatment effects has suggested that expectation processes are crucially involved in the underlying mechanism (Petrovic et al., 2010; Büchel et al., 2014). For any given treatment, expectations about its effect will be built up in the subject or the patient. Verbal information about the effectiveness of the treatment is one source of information affecting the expectations. Other contextual factors in treatment may also change the expectations—including how invasive the treatment is (e.g., injections seems to be more effective than giving a pill). Importantly, also low-level conditioning effects are important for determining the expectation effect (Amanzio and Benedetti, 1999; Jensen et al., 2012, 2015), albeit in lower levels of the hierarchical network. Thus, placebo effects are not dependent on conscious mechanism. In more formal terms all these sources of information processing change the priors of the brain in different hierarchical levels, all of which are thought to contribute to the placebo effect.

The underlying neural mechanism of the placebo effect has mostly been studies with regards to pain, where also the underlying opioid system has been proposed of importance (Petrovic et al., 2002; Zubieta et al., 2005; Wager et al., 2007). Further, placebo treatment has been suggested to change the neural processing underlying emotions (Petrovic et al., 2005) depression (Mayberg et al., 2002) and Parkinson’s disease (de la Fuente-Fernández et al., 2001). Moreover, similar manipulations of expectations have been shown to change how visual stimuli are processed (Sterzer et al., 2008; Schmack et al., 2013) in line with the idea that any type of perception is perceived in relation to the expectations of the systems.

How profoundly can expectation change the experience of the world? It has been suggested that extremely powerful priors are essentially the cause of hallucinations and delusions in psychosis (Frith, 2007; Fletcher and Frith, 2009; Adams et al., 2013). In line with this idea, manipulations of higher order expectations have shown that delusion prone individuals will experience the world more in line with those expectations (Schmack et al., 2013; Teufel et al., 2015).

A common theme, apart from the involvement of expectations, is dopamine system involvement in different aspects of placebo responses (de la Fuente-Fernández et al., 2001; Scott et al., 2008). As a main contributor also in psychosis, it may have a specific role in the balance between priors and observation.

Thus, in a predictive coding framework, priors change the way information is processed, even to the extent that delusions may arise in realms beyond control and awareness. Moreover, certain personality traits, such as delusion proneness, may explain why some individuals are more likely to develop pathogenic priors.

Interestingly, it has furthermore been suggested that functional sensory or motor symptoms in somatization and conversion disorder may be initiated and maintained by strong, although not necessarily conscious, priors (Edwards et al., 2012). In the sensory domain, the results of strong priors are well formed precepts, which may or may not be accurate representations of the world. In the motor domain, strong priors will elicit motor behavior, or its absence, through top-down influence on motor reflex arcs which, involuntarily generated, is perceived as abnormal behavior and symptoms of a neurological disorder.

The interoceptive system has been proposed to be likewise affected by expectation (Barrett and Simmons, 2015). Thus, the experienced bodily state will be determined not only by input from different interoceptive channels but also by expectations regarding the state itself. In particular, homeostatic cues from the hormonal, immune, metabolic and autonomic nervous systems have been suggested to generate error signals resulting in either bottom-up adjustment of predictions, or, top-down influence over physiological homeostasis (Seth, 2013; Barrett and Simmons, 2015). Thus, by predictive coding, the brain not only acquires and adjusts to homeostatically relevant information; it also orchestrates the adaption of the organism in relation to physiological needs instructed by priors. In particular, according to the model, the latter occurs in parts of the system where priors are strong and observations weak or imprecise, a balance likely to be of importance in homeostatic systems relying on fixed parameters to maintain the organism within a physiological state.

It is hypothesized (Seth, 2013; Barrett and Simmons, 2015) that not only basic homeostasis—converging particularly on the anterior insular cortex (AIC)—but also higher order self-related representations of emotion, agency, self-narration and body-ownership—subserved in ACC, posterior ventromedial PFC, posterior OFC—and supporting conscious self-perception and attentional control, are implicated in a wider interoceptive predictive coding system instantiated by the brain. Through the multisensory representation of in particular homeostatically relevant predictions distributed in the AIC and ACC, modulation of attentional, sensory and behavioral responses, is attained and transmitted in the wider system. Through these channels higher order functions may be recruited in minimizing prediction error by adjusting priors or instructing behavior on the conscious level.

Thus, by engaging physiological control mechanisms at the core of the organism, as well as attentional, sensory and behavioral responses also under the influence of higher cognitive processing, a powerful and integrated system answering to ecological needs of the organism is running, orchestrated within a predictive coding framework, in the brain.

Here we propose that a multitude of factors—psychological and or physical trauma, helplessness and hopelessness, familial expectations and obligations, the predicaments of migration and asylum seeking, including negative expectations regarding chances of obtaining a PRP—are crucial for setting inner priors of the interoceptive system to extreme levels in predisposed individuals. These priors may include (conscious or non-conscious) models untenable for bodily function under massive external stress thus eliciting a vulnerable state evolving into RS.

A situation of extreme stress and negative prospects (nocebo) is under normal circumstances not detrimental as interoceptive and exteroceptive input generates prediction errors driving physiological and behavioral change aimed at overcoming the situation. On the contrary, when strong priors are set “low”, due to previous experience projecting to the present, the same nocebo situation will only accord with the predictions and adjustment will be absent thus perpetuating negative predictions with corresponding physiological, attentional, sensory and behavioral consequences.

Moreover, even if the interoceptive and exteroceptive input improve, prediction error may—provided priors are sufficiently strong, or sensory input imprecise—drive adjustment not of the model, but instead towards the prediction, in which case homeostatic mechanisms are directed top-down to attain a physiological state in correspondence with the prediction so as to minimize prediction error. The corresponding percept of the internal state represented in the AIC and the ACC is through the wider system responsible for modulation of cognitive, emotive and behavioral processes thus unlocking the full potential of the organism’s predictive capacity resulting also in a mind-set corresponding to the prediction.

At this stage, negative predictions having generalized in higher and lower levels of the hierarchy, and physiological systems threatening homeostasis, the organism, at some point, adopts a behavior which elicits support from its surroundings; an idiom of distress.

Within a predictive coding framework this may be interpreted either as an attempt at minimizing prediction error by projecting the interocepted state onto the world as to affect it to accord with the prediction, in which case the intended result is the prevention of help from the surrounding; or, as a change in strategy and if so presumably driven by another set of priors corresponding to a rescue attempt from an inexorably escalating development. Interestingly, Seth (2013) finds support for an extended Bayesian framework encompassing also social interaction. Drawing on evidence from psychosis, behavior such as loss or alteration of general function may be as powerful as a delusion. Importantly, the response from the surrounding may on this hypothesis not only contribute to perpetuation of an illness-state but also possibly hold the key to its resolve.

We thus propose that RS may be conceived within a predictive coding framework, as a condition where predisposing and contextual factors generate in negative expectations and beliefs instantiated in fixed priors, which drives homeostatic and behavioral effects as well as self-perception, towards the prediction, minimizing prediction error, however at the cost of pushing the physiological, cognitive and emotional state further away from that which sustains life. The resulting behavior—described in terms of apathy, RS or catatonia—may be interpreted as, an outwardly broadcasted self-representation functioning as to minimize prediction error by extending also into the world the interocepted state in order to affect it accordingly, or, as a behavior serving to elicit support from the surrounding. In either case, the particular behavior, intended for a specific purpose, is conceivably one corresponding to culturally sanctioned expectations of what that behavior entails. Consequently, culture-bound reaction patterns are predicted by the model.

## Discussion

With regards to the phenomenon referred to as RS, our analysis has suggested catatonia to supply the best fit with clinical data, culture-bound psychogenesis to account for the regional distribution and predictive coding to supply a promising context in which to express a mechanistic model. We have purposely omitted to develop an account of the neural components instantiating the Bayesian machinery in the brain and instead direct the reader to recent conceptualizations (Edwards et al., 2012; Seth, 2013; Barrett and Simmons, 2015).

Our analysis has lead us to a proposal that catatonia in certain instances may be culture-bound, which, considering the organic presentation of the disorder and its historical relation to schizophrenia, is highly controversial. Nevertheless, the current conception of catatonia as a neuropsychiatric syndrome associated with systemic illness (Fink et al., 2015) implies the possibility of a heterogeneous etiology.

Relatedly, an analysis of catatonia and Parkinson’s disease—conceived as movement disorders—at the level of the brain, has been suggested should invoke a “principle of double way”, asserting that “function of the same anatomical apparatus may be disturbed by both organic lesions and psychological alterations”. Hypothetically, the same motor loop may be abnormally affected either by psychological (cortical) top-down regulation, or, by aberrant (subcortical) bottom-up regulation illustrated by akinetic catatonia and Parkinson’s disease functionally affecting the same “motor loop”, however, by different mechanisms originating in the orbitofrontal cortex (OFC) and the substantia nigra respectively (Northoff, 2002). Even though this proposal is not set in a predictive coding framework, it may be reinterpreted in relation to motor predictions and proprioceptive input generating error signals eliciting top-down directed (absence of) movement or bottom-up adjustments of predictions, both converging on intermediate levels of the hierarchy and eliciting motor symptoms in accordance with a recent proposal by Edwards et al. (2012).

Catatonia has further been conceived of as a disorder resulting from abnormal emotional processing. Catatonic patients (*n* = 10) exhibited altered activity in mOFC and mPFC as well as abnormal orbitofrontal and premotor/motor cortical functional connectivity on exposure to negative emotional images and compared to psychiatric and healthy controls. Also, catatonic behavioral and affective symptoms correlated with deactivation in OFC whereas motor symptoms correlated with mPFC activation (Northoff et al., 2004). It was suggested that abnormal emotional processing and deactivation of OFC—through connections to the basal nucleus of amygdala implicated in affective inhibition by cognitive control—result in subsequent altered activity in medial prefrontal and premotor/motor function generating affective, behavioral and motor symptoms of catatonia. Response to anxiolytic drugs and self-report of overwhelming uncontrollable emotions—both notably reported also in RS (Bodegård, 2005b; Engström, 2013)—is taken to support the hypothesis (Northoff et al., 2004). Supplying an adequate fit with the data, this hypothesis nevertheless lacks in sufficient precision to allow anything but a very general reconceptualization within a predictive coding framework amounting to an analysis involving inadequate priors generating prediction errors the system adapts to by actions on intermediate levels involving lower as well as higher processing nevertheless below the conscious level.

An alternate exploration of catatonia recognizes connexion with anxiety or fear states on the basis of immediate treatment response to anxiolytics drugs, taken to support a limbic system pathophysiology (Daniels, 2009). Deficits in akinetic catatonia, such as stupor, mutism and negativism, are however consistent also with an underlying motivational deficit suggesting that suppression of incentive salience (“wanting”; Berridge and Robinson, 2003), mediated in dopaminergic mesolimbic structures projecting into prefrontal areas could account for core symptoms. Dopamine, of importance in placebo (Scott et al., 2008) and delusions (Adams et al., 2013) may interplay in shifting the balance between prior and observation also generating catatonic symptoms in relation to predictions.

Other than reconceptualizations of previous findings complying with a Bayesian analysis, there are clinical evidence of a multifactorial genesis involving also psychogenic generation of catatonia. Case-reports attributing catatonia to conversion (Jensen, 1984; Shah et al., 2012) and catatonia (*n* = 10) as well as conversion (*n* = 5) successfully confirmed through a common method of Amytal interviewing (Iserson, 1980) are found in support. Moreover, catatonic presentation varying throughout history (Shorter, 2006, 2012) attributes to it a characteristic generally considered a hallmark of conversion disorder and Shorter (2012) asserts “It is important to understand that cultural suggestion can cause patients to present catatonic symptoms in some epochs, but not in others, whereas changes in diagnostic fashion determine whether physicians make the diagnosis or not”. Thus, it does not seem unlikely that the brain, influenced by “cultural suggestion”, could generate catatonic symptoms and the model here proposed supply a mechanistic account of how this could be instantiated.

Pertaining to RS, there are, as we have here demonstrated, indications that mandate the assertion equating RS with catatonia. Apart from clinical characteristics, in particular reports of normalization in response to midazolam, implies this hypothesis should be evaluated and we have suggested means thereto. As for the regional distribution, considered as a hypothesis, it is difficult to test. In different parts of science, however, different truth-criteria are manifest and from the general point of view, broader involvement in this issue, and in particular an epidemiological effort, is much needed.

Other than treatment already routinely offered in catatonia—which is reported safe and efficient, also in children adolescents—our model predicts no magic bullet. On the contrary, if fixed predictions—laid down by previous experience projecting into the present, in order to tell the future—generate in prediction error denial, with subsequent drive in homeostatic and higher level systems, towards the perpetuation of those malicious predictions, by arranging the inner and outer world so as to accord therewith, the situation indeed seems desperate.

Nevertheless, on the hypothesis that the behavior characterizing RS is a social extension of interoceptive predictions, which serves to either sound the alarm or perpetuate inappropriate priors, the behavioral pattern represents on some level a strategy selected in a social context. (Which is not to say it is in any way voluntary). If this line of reasoning is correct, which indeed is implied by the phenomenon respecting barriers pertaining to language, culture, ethnicity and national borders, measures aimed at pre-empting the unfortunate strategy should be enforced. Certainly, a deepened understanding of the history, culture and situation of risk groups individuals would be necessary in order to reach out to these individuals.

The appeal to culture-bound psychopathology raises an ethical dilemma. The argument we have presented, according to which cultural sanctioning contributes to the generation of specific kinds of behavioral patterns, implies that by offering treatment, to which there is no alternative, we are also, on another level, causing new cases.

## Conclusion

The regional distribution and the prevalence of RS are challenging to explain. Firstly, we have tried to establish that RS represents a disorder previously described. Historical accounts demonstrate that so is the case. We find no reason to ascribe to this phenomenon a novel diagnostic entity.

Secondly, bearing this in mind, the diagnostic fit to known disorders and hypotheses previously put forward have been evaluated. We have argued catatonia to supply the best fit and suggested means of examining this hypothesis in accordance with clinical practise and by neuroimaging. Catatonia, recently reconceptualized, amounts to a phenomenological description of a clinical entity for which there presumably can be different causes.

Thirdly, the regional distribution, we have argued, is best explained by perceiving RS as culture-bound. Importantly, this does not preclude other factors to interplay in pathogenesis. On the contrary, individual predisposition, traumatization, contextual factors as well as culturally sanctioned beliefs and expectations, may all be involved.

Lastly, we have provided a predictive coding model of RS. On the basis of extreme priors, fixed by previous experiences, the percept of the inner and outer world is stable and skewed. Consequently, error signal minimization is directed towards effecting the inner and outer worlds to accord with the predictions which unharnesses homeostatic and behavioral responses with that objective. This includes, on a social extension, the projection of a culturally sanctioned idiom of distress also interpretable in a predictive coding framework. Accommodating an extensive multilevel involvement of homeostatic, cognitive and emotional systems with deep impact on behavior influenced by cultural expectations, this analysis is compatible with RS being catatonia, culture-bound.

## Author Contributions

KS: wrote manuscript, responsible of general ideas. PP: contributed in revising. PP and other authors: commented on previous versions of manuscript, helped developing lines of argument.

## Conflict of Interest Statement

The authors declare that the research was conducted in the absence of any commercial or financial relationships that could be construed as a potential conflict of interest.

## Footnotes

^1^Under the slow intravenous injection of amobarbital, continuous interviewing and evaluation of symptoms, patients with psychogenic impairment of consciousness exhibit symptom relief.

^2^A modern version of the Amytal interview in which lorazepam or diazepam is used.
